# Pericytes mediate neuroinflammation via Fli-1 in endotoxemia and sepsis in mice

**DOI:** 10.1007/s00011-025-02000-z

**Published:** 2025-01-25

**Authors:** Pengfei Li, Liu Liu, Perry V. Halushka, Maria Trojanowska, Guirong Wang, Adviye Ergul, Hongkuan Fan

**Affiliations:** 1 Department of Pathology and Laboratory Medicine, Medical University of South Carolina, 173 Ashley Ave, Charleston, SC 29425 USA; 2 Department of Medicine, Medical University of South Carolina, Charleston, SC 29425 USA; 3 Arthritis and Autoimmune Diseases Center, Boston University, Boston, MA 02118 USA; 4 Departments of Surgery and Microbiology and Immunology, SUNY Upstate Medical University, Syracuse, NY 13210 USA

**Keywords:** Fli-1, Pericytes, Neuroinflammation, Sepsis

## Abstract

**Background:**

Sepsis-associated encephalopathy (SAE) often results from neuroinflammation. Recent studies have shown that brain platelet-derived growth factor receptor β (PDGFRβ) cells, including pericytes, may act as early sensors of infection by secreting monocyte chemoattractant protein-1 (MCP-1), which transmits inflammatory signals to the central nervous system. The erythroblast transformation-specific (ETS) transcription factor Friend leukemia virus integration 1 (Fli-1) plays a critical role in inflammation by regulating the expression of key cytokines, including MCP-1. However, the role of pericyte Fli-1 in neuroinflammation during sepsis remains largely unknown.

**Methods:**

WT and pericyte-specific *Fli-1* knockout mice were subjected to endotoxemia through LPS injection or sepsis via cecal ligation and puncture (CLP). In vitro, *Fli-1* was knocked down using small interfering RNA in cultured mouse brain pericytes, followed by LPS stimulation.

**Results:**

Elevated *Fli-1* levels were observed in isolated brain pericytes 2 h after LPS administration, in brain tissues 4 h after CLP, and in cultured mouse brain pericytes 2 h after LPS stimulation in vitro. In endotoxemic mice, pericyte-specific *Fli-1* knockout reduced expression of MCP-1 and IL-6 in brain tissue 2 h after LPS injection. At 24 h post-LPS administration, protein levels of MCP-1 and IL-6, and microglia activation were suppressed in pericyte-*Fli-1* knockout mice. Additionally, *Fli-1* deficiency in pericytes significantly reduced MCP-1 and IL-6 mRNA levels in the brain tissue 4 h after CLP. Moreover, in cultured brain pericytes, *Fli-1* knockdown markedly decreased MCP-1 and IL-6 levels after LPS stimulation. Notably, LPS stimulation increased *Fli-1* levels via TLR4-Myd88 signaling, which subsequently led to elevated production of MCP-1 in brain pericytes.

**Conclusions:**

*Fli-1* in pericytes may serve as a crucial mediator of neuroinflammation during sepsis by directly regulating pivotal cytokines such as MCP-1 and IL-6. Therefore, Fli-1 has the potential to serve as a therapeutic target in SAE and other neuroinflammatory disorders.

## Introduction

Sepsis is the leading cause of mortality and critical illness in intensive care unit patients, characterized by a systemic inflammatory response [1–5]. Sepsis-associated encephalopathy (SAE) affects approximately 20–70% of septic patients and significantly contributes to increased mortality following sepsis [5–8]. Although the precise pathophysiology of SAE is not fully understood, neuroinflammation is recognized as a key factor [5, 8]. Neuroinflammation involves the activation of immune cells and elevated levels of inflammatory mediators, such as monocyte chemoattractant protein-1 (MCP-1) and IL-6, in the brain [8–10]. Recent studies have shown that brain platelet-derived growth factor receptor β (PDGFRβ) cells, including pericytes, contribute to the early neuroinflammatory response by rapidly relaying inflammatory signals to neurons through MCP-1 production within 2 h of systemic inflammation [9, 11]. In the early stages of neuroinflammation, microgliosis and astrogliosis have not been observed [9, 11], suggesting that pericytes may play a crucial role in the initial neuroinflammatory response.

Pericytes line the endothelial cells of all microvessels throughout the body. They are characterized by the expression of markers such as PDGFRβ and neural/glial antigen 2 (NG2) [12, 13]. Pericytes are involved in key biological processes, including blood vessel stabilization, blood-brain barrier (BBB) regulation, and blood flow control [12, 13]. Increasing evidence suggests that pericytes also play a critical role in regulating inflammation across various diseases, including sepsis, Alzheimer’s disease (AD), and cancer [9, 13–16]. They mediate inflammation by recruiting leukocytes, modulating endothelial function, interacting with immune cells, and producing inflammatory mediators such as MCP-1 and IL-6 [9, 13–16]. Pericytes have been implicated in mediating lung inflammation during sepsis [9, 16, 17]. Our previous studies highlighted that activated lung pericytes contribute to sepsis-induced inflammation via Friend leukemia virus integration 1 (*Fli-1*) [9]. However, the involvement of brain pericytes in the early phase of neuroinflammation during sepsis has yet to be thoroughly investigated.

Fli-1, an ETS transcription factor, regulates a broad range of biological processes, including cancer development, fibrosis, vasculopathy, and inflammation [18–23]. Fli-1 is expressed in endothelial cells, macrophages, B cells, T cells, and pericytes, and it regulates the expression of key cytokines such as MCP-1 and IL-6 [9, 12, 21, 22]. Our previous study identified *Fli-1* as a crucial regulator of inflammation in lung pericytes. Specifically, *Fli-1* knockout in these cells attenuated the inflammatory response, reduced vascular leakage, and decreased mortality in a murine sepsis model [9]. We have also observed elevated levels of *Fli-1* in brain pericytes and found that Fli-1 inhibition can attenuate neuroinflammation in AD [14]. However, the role of pericyte *Fli-1* in neuroinflammation during sepsis remains largely unknown.

These findings led us to investigate the role of pericyte *Fli-1* in the early phase of neuroinflammation during sepsis. We hypothesize that pericytes mediate the early neuroinflammatory response in sepsis through *Fli-1* and that *Fli-1*-mediated pericyte activation contributes to this response by producing MCP-1.

## Materials and methods

### Generation of pericyte-specific *Fli-1* knockout mice

Pericyte-specific *Fli-1* knockout mice were generated by crossing PDGFRβ-P2A-CreER^T2^ mice (Stock No: 030201, The Jackson Laboratory) with *Fli-1*^floxp/floxp^ mice (provided by Dr. Maria Trojanowska, Boston University). Mouse genotyping analysis was performed by polymerase chain reaction (PCR) using DreamTaq DNA Polymerase (#EP0705; Thermo Fisher Scientific).

### Lipopolysaccharide-induced endotoxemia

All procedures complied with the standards for the care and use of animal subjects as stated in the *Guide for the Care and Use of Laboratory Animals* (Institute of Laboratory Resources, National Academy of Sciences, MD). The protocol for all animal studies was approved by the Institutional Animal Care and Use Committee at the Medical University of South Carolina. Endotoxemia was induced in WT mice and pericyte-specific *Fli-1* knockout mice (2–4 months old; male and female) via intraperitoneal injection of LPS (15 mg/kg body weight; *E. coli* LPS 0111: B4; Sigma-Aldrich) dissolved in sterile saline. Mice were randomly assigned to one of four groups: (1) WT mice receiving sterile saline; (2) Pericyte-specific *Fli-1* knockout mice receiving sterile saline; (3) WT mice receiving 15 mg/kg body weight of LPS; or (4) Pericyte-specific *Fli-1* knockout mice receiving 15 mg/kg body weight of LPS. Mice were sacrificed at 2–24 h post LPS injection, and brain tissues were collected for analysis.

### Mouse brain pericyte isolation

Mouse brain pericytes were isolated from WT and pericyte-specific *Fli-1* knockout mice (2 months old; male and female) as described previously [24]. Briefly, single-cell preparations from whole brain digests were negatively selected by CD31, O4, CD11b, and ACSA-2 magnetic microbeads (#130-097-418, # 130-096-670, #130-049-601, #130-123-284; Miltenyi Biotec Inc.) to deplete endothelial cells, oligodendrocyte, microglia cells, and astrocytes, respectively. Pericytes were labeled with PE-conjugated anti-CD13 (#558745, BD Pharmingen™) and anti-PE magnetic microbeads (#130-048-801, Miltenyi Biotec Inc.) and isolated using magnetized columns. CD13-positive brain pericytes were cultured in pericyte medium supplemented with pericyte growth supplement, 2% fetal bovine serum, and 1% penicillin/streptomycin (#1231, ScienCell Research Laboratories). *Fli-1* and MCP-1 mRNA levels were measured by real-time PCR. In a separate experiment, endotoxemia was induced in WT mice (2–4 months old; male and female) by intraperitoneal injection of LPS (15 mg/kg body weight; *E. coli* LPS 0111:B4; # L2630, Sigma-Aldrich) dissolved in sterile saline. The mice were randomly assigned to one of two groups: (1) WT mice receiving sterile saline or (2) WT mice receiving 15 mg/kg body weight of LPS. At 2 h post-injection, brain pericytes were isolated as described, and *Fli-1* protein levels were measured by Western blot in these freshly isolated cells.

### Cecal ligation and puncture-induced sepsis

All procedures complied with the standards for the care and use of animal subjects as stated in the *Guide for the Care and Use of Laboratory Animals* (Institute of Laboratory Resources, National Academy of Sciences, MD). The protocol for all animal studies was approved by the Institutional Animal Care and Use Committee at the Medical University of South Carolina. All surgery was performed under anesthesia. CLP was performed on WT and pericyte-specific *Fli-1* knockout mice (2–4 months old; male and female) as described previously [9]. Briefly, the cecum was ligated at the colon juncture and punctured twice with a 22-gauge needle, followed by subcutaneous fluid resuscitation with sterile saline. A sham operation was performed without cecum ligation and puncture. Mice were sacrificed 4 h post-CLP, and brain tissues were collected for further analysis.

### Mouse brain pericyte culture and stimulation

Mouse brain pericytes were purchased from ScienCell Research Laboratories and cultured in pericyte medium, supplemented with pericyte growth supplement, 2% fetal bovine serum, and 1% penicillin/streptomycin (#1231, ScienCell Research Laboratories). Cells were transfected with *Fli-1*-specific small interfering RNA (siRNA, #4390771, AMBION) or control siRNA using HiPerFect Transfection Reagent (#301704, QIAGEN) for 24 h, followed by LPS stimulation (200 ng/mL) for 2–24 h. Total RNA and supernatants were collected for further analysis. In a separate experiment, pericytes were transfected with TLR4 (#AM16708-75404, AMBION) or MyD88 siRNA (#AM16708-156408, AMBION) or control siRNA using HiPerFect Transfection Reagent (#301704, QIAGEN) for 24 h, followed by LPS stimulation (200 ng/mL) for 2 h. Total RNA and supernatants were collected for analysis.

### Real-time reverse transcription-polymerase chain reaction (RT-PCR)

Total RNA was extracted from brain tissues and brain pericytes using RNeasy plus mini kit (#74134, Qiagen). cDNA was synthesized with a High-Capacity cDNA Reverse Transcription Kit (#4368814, ThermoFisher Scientific). Quantitative real-time PCR was performed using Prism 7300 Real-Time PCR System (Applied Biosystems) with SYBR Green PCR Kit (#330629, Qiagen) in a final reaction volume of 25 µl. Data were analyzed with 2^−ΔΔCt^ value calculation using GAPDH for normalization.

### ELISA

The levels of MCP-1 and IL-6 were measured using an MCP-1 or IL-6 Mouse ELISA Kit (#BMS6005, #KMC0061; ThermoFisher Scientific), following the manufacturer’s instructions.

### Western blotting analysis

Isolated mouse brain pericytes were lysed with ice-cold RIPA lysis buffer (#9806, Cell Signaling). Western blot was performed as described [9]. All lysed samples were kept on ice for 30 min and centrifuged for 10 min at 4 °C at 12,000 g. The supernatant was collected and stored at -20 °C until further analysis. Cell lysates were subjected to 10% SDS-PAGE and transferred onto a polyvinylidene difluoride membrane. The membranes were blocked with 7% milk in TBST (20 mM Tris, 500 mM NaCl, and 0.1% Tween 20) for 1 h. Then, membranes were incubated with primary antibody overnight at 4 °C. Fli-1 primary antibody was from Abcam (ab124791). GAPDH primary antibody (#5174) was from Cell Signaling. The membranes were washed three times with TBST and incubated with HRP-conjugated secondary antibody in a blocking buffer for 1 h. After washing three times with TBST, immunoreactive bands were visualized by incubation with ECL plus detection reagents (#A38554, ThermoFisher Scientific). The densitometry of bands was quantified with Image J2 software.

### Immunocytochemistry

Frozen mouse brain tissues were cut into 10 μm sections, fixed with 4% paraformaldehyde (PFA) for 15 min at room temperature, and then washed with PBS. The sections were then incubated in blocking buffer followed by primary antibody Iba1 (#ab178846, Abcam) overnight at 4 °C. Sections were washed three times with PBS and incubated with Alexa Fluor 647 donkey anti-rabbit IgG (H + L) secondary antibody (#A31573, ThermoFisher Scientific) diluted 1:500 in PBS for 1 h at room temperature. For analysis, four images at 40× objective were taken randomly from each mouse using a SP8 confocal microscopy, and the fluorescence intensity in each acquired image was analyzed in the NIH ImageJ software.

### Data analyses

Data are presented as means ± standard error of the mean (SEM). Statistical significance was determined by analysis of variance (ANOVA) or Student’s *t*-test using GraphPad Prism software. A value of *p* < 0.05 was considered statistically significant.

## Results

### Knockout of *Fli-1* in pericytes reduces brain inflammation in LPS-induced endotoxemic mice

**Fig. 1:**
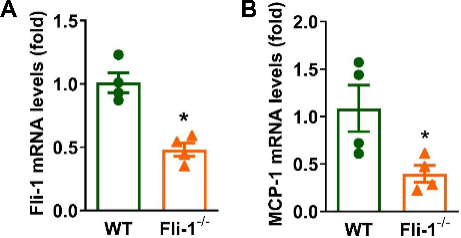
Reduced expression of *Fli-1* and MCP-1 in brain pericytes from pericyte-specific *Fli-1* knockout mice. Brain pericytes were isolated from wild-type (WT) and pericyte-specific Fli-1 knockout mice using magnetic microbeads. The mRNA levels of *Fli-1* (**A**) and MCP-1 (**B**) in these cells were quantified by Real-time PCR. *N* = 4 mice per group. Data are presented as means ± SE. Statistical significance was determined using Student’s t-test, with *p < 0.05 indicating a significant difference compared to the WT group

**Fig. 2:**
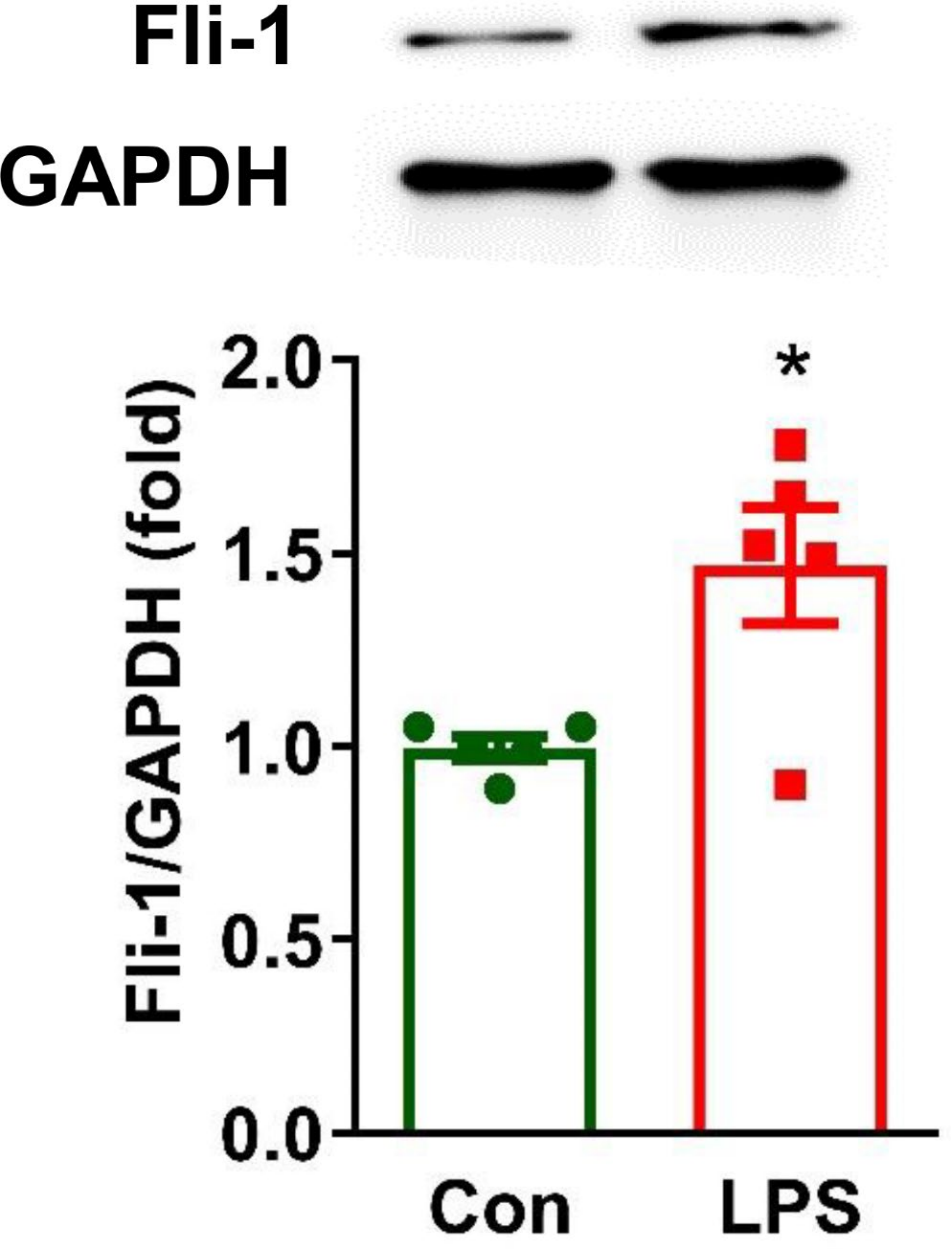
Increased *Fli-1* protein levels in brain pericytes from LPS-induced endotoxemic mice. Endotoxemia was induced in wild-type (WT) mice through an intraperitoneal injection of LPS (15 mg/kg body weight) in sterile saline. Brain pericytes were isolated from both WT and endotoxemic mice 2 h post-LPS administration using magnetic microbeads. *Fli-1* protein levels in these cells were assessed by Western blot. *N* = 5 (2 mice were used to get enough cells for each group for 1 data point). Data are presented as means ± SE. Statistical significance was determined using Student’s t-test, with *p < 0.05 indicating a significant difference compared to the Control group

**Fig. 3:**
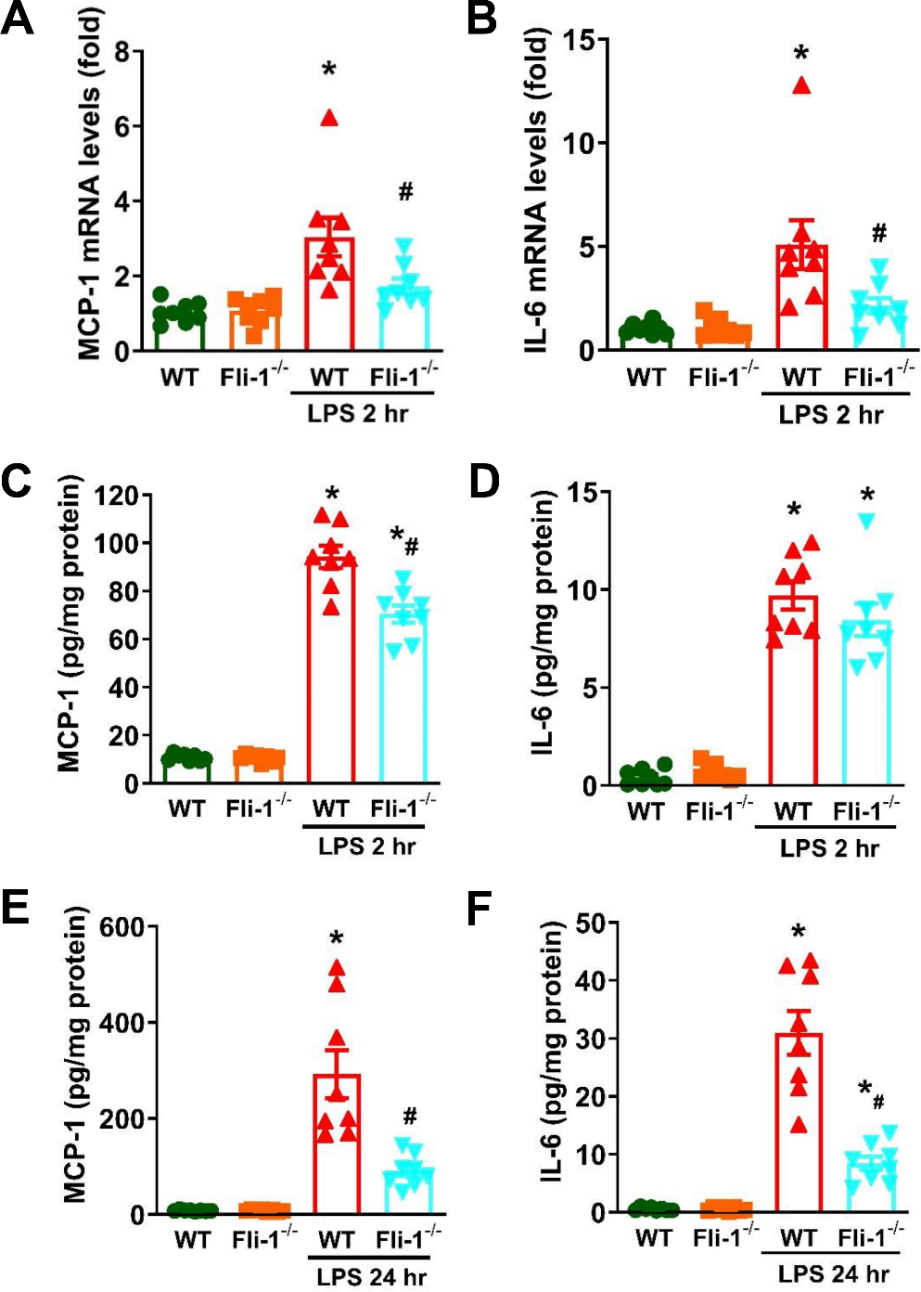
Pericyte-*Fli-1* knockout reduces brain inflammatory response in LPS-induced endotoxemic mice. Endotoxemia was induced in wild-type (WT) and pericyte-specific *Fli-1* knockout mice by intraperitoneal injection of LPS (15 mg/kg body weight) dissolved in sterile saline. Brain tissues were collected at 2–24 h post-LPS administration. The mRNA levels of MCP-1 (**A**) and IL-6 (**B**) were measured in brain tissues 2 h post-LPS by Real-time PCR. Protein levels of MCP-1 (**C**) and IL-6 (**D**) were assessed in brain tissues 2 h post-LPS using ELISA. MCP-1 (**E**) and IL-6 (**F**) levels in brain tissues were also measured 24 h post-LPS by ELISA. *N* = 8 mice per group. Data are presented as means ± SE. *p < 0.05 indicates a significant difference compared to the WT group, while #*p* < 0.05 indicates a significant difference compared to the WT + LPS group, both determined by ANOVA

**Fig. 4:**
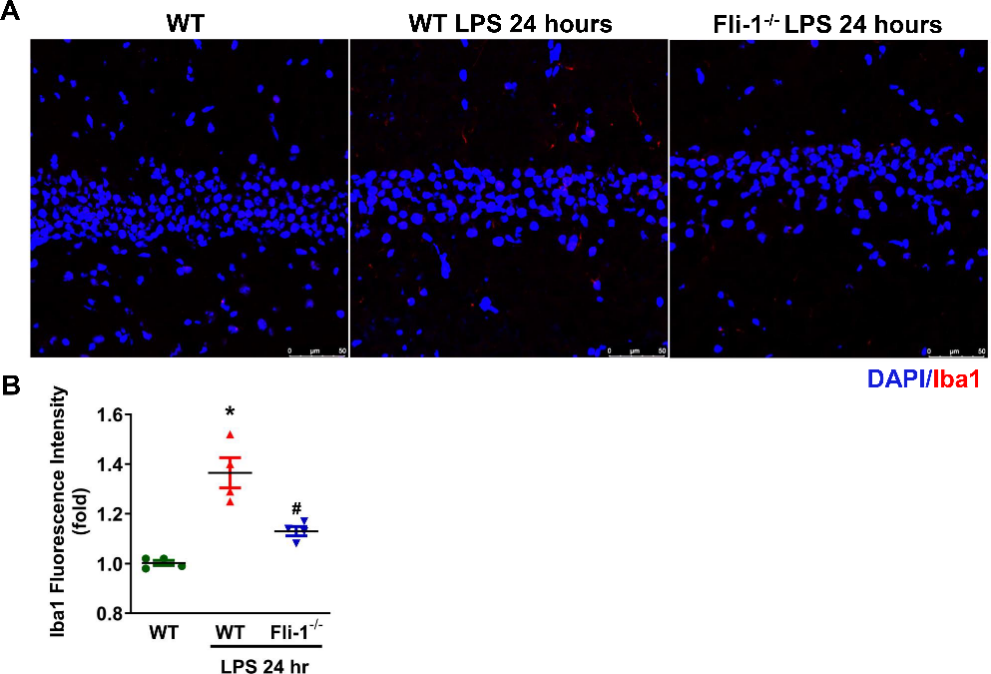
Pericyte-*Fli-1* knockout reduces brain microglia activation in LPS-induced endotoxemic mice. Endotoxemia was induced in wild-type (WT) and pericyte-specific *Fli-1* knockout mice by intraperitoneal injection of LPS (15 mg/kg body weight) dissolved in sterile saline. Brain tissues were collected at 24 h post-LPS administration. **A** Representative fluorescence images of brain tissue stained for Iba1 (red) and nuclei (DAPI, blue). Scale bar: 50 μm. **B** Quantification analysis of Iba1 fluorescence intensity was performed; *n* = 16 random fields from four mice/group. *p < 0.05 indicates a significant difference compared to the WT group, while #*p* < 0.05 indicates a significant difference compared to the WT + LPS group, both determined by ANOVA

To investigate the role of pericyte *Fli-1* in brain inflammation, we generated tamoxifen-induced pericyte-*Fli-1* knockout mice. As shown in Fig. 1A, *Fli-1* mRNA levels were significantly reduced in isolated brain pericytes from these knockout mice compared to WT mice. Basal MCP-1 mRNA levels were also lower in *Fli-1* knockout pericytes (Fig. 1B). In LPS-induced endotoxemic mice, *Fli-1* protein levels were significantly elevated in brain pericytes 2 h after LPS administration compared to the control group (Fig. 2). Additionally, mRNA levels of MCP-1 and IL-6 were markedly increased in brain tissues 2 h after LPS administration, which were significantly reduced in pericyte-*Fli-1* knockout mice (Fig. 3A-B). Protein levels of MCP-1 and IL-6 were also elevated in the brain at this time (Fig. 3C-D). While pericyte-specific *Fli-1* knockout significantly reduced MCP-1 levels (Fig. 3C), it led to a non-significant decrease in IL-6 levels (Fig. 3D). Furthermore, *Fli-1* knockout in pericytes decreased both MCP-1 and IL-6 protein levels in the brain tissue of endotoxemic mice 24 h after LPS administration (Fig. 3E-F). Additionally, microglial activation was assessed through immunostaining with Iba1, and LPS treatment significantly enhanced microglial activation in the brain. However, pericyte-specific Fli-1 knockout notably attenuated this activation (Fig. 4).

### Knockout of Fli-1 in pericytes reduces brain inflammation in CLP-induced septic mice

**Fig. 5:**
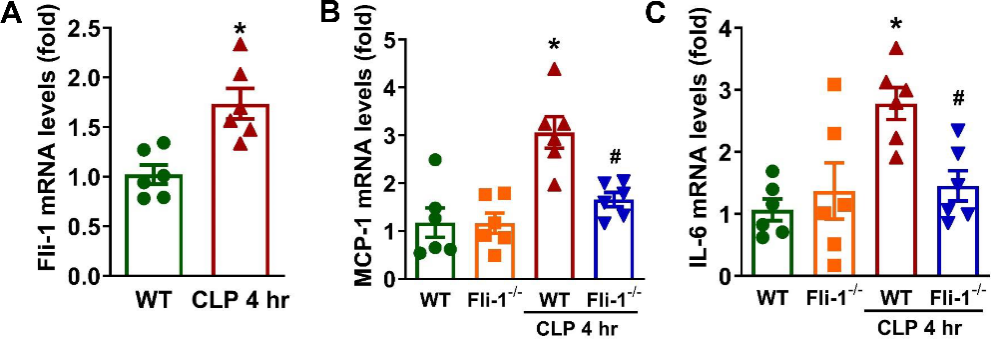
Pericyte-*Fli-1* knockout mitigates brain inflammation in cecal ligation and puncture-induced septic mice. Wild-type (WT) and pericyte-*Fli-1* knockout mice were subjected to sham or cecal ligation and puncture (CLP) surgery. Brain tissues were collected 4 h post-CLP. **A***Fli-1* mRNA levels in the brain were measured by Real-time PCR. *N* = 6 mice per group. Data are presented as means ± SE. Statistical significance was determined using Student’s t-test, with *p < 0.05 indicating a significant difference compared to the WT-sham group. The expression levels of MCP-1 (**B**) and IL-6 (**C**) were also determined by Real-time PCR. *N* = 6 mice per group. Data are presented as means ± SE. *p < 0.05 indicates a significant difference compared to the WT-sham group, while #*p* < 0.05 indicates a significant difference compared to the WT-CLP group, both determined by ANOVA

To further confirm the role of *Fli-1* in brain inflammation during sepsis, CLP was performed in both WT mice and pericyte-*Fli-1* knockout mice. *Fli-1* mRNA levels were significantly elevated in the brain tissue of septic mice 4 h post-CLP compared to sham-operated mice (Fig. 5A). Additionally, mRNA levels of MCP-1 and IL-6 were significantly increased in the brain tissue of septic mice at 4 h post-CLP, but these levels were reduced in pericyte-*Fli-1* knockout mice (Fig. 5B-C).

### *Fli-1* regulates LPS-induced early inflammatory response in cultured brain pericytes

**Fig. 6:**
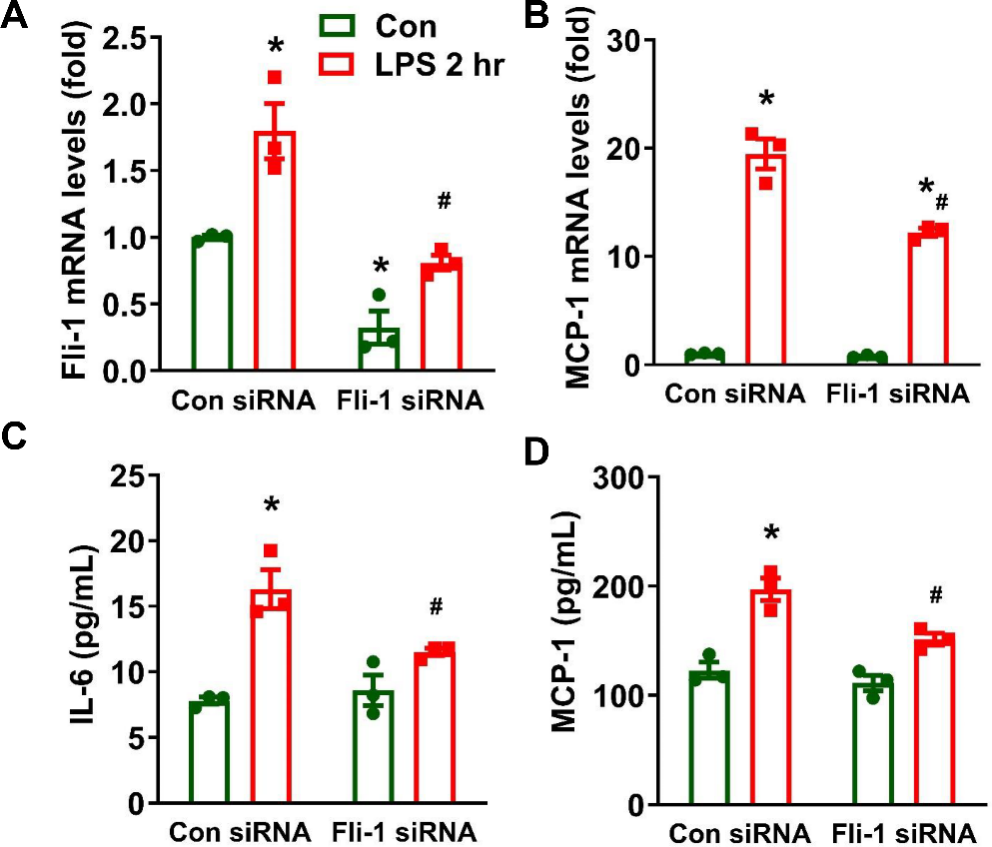
Knockdown of *Fli-1* reduces LPS-induced early inflammatory response in cultured mouse brain pericytes. Cells were transfected with Fli-1-specific small interfering RNA (siRNA) or control siRNA for 24 h, followed by LPS stimulation (200 ng/mL) for 2 h. mRNA levels of *Fli-1* (**A**) and MCP-1 (**B**) were measured by Real-time PCR. IL-6 (**C**) and MCP-1 (**D**) levels in the supernatants were quantified by ELISA. *N* = 3 independent experiments. Data are presented as means ± SE. Statistical significance was determined using ANOVA, with *p < 0.05 indicating a significant difference compared to the Control siRNA group and #*p* < 0.05 indicating a significant difference compared to the Control siRNA + LPS group

To investigate the role of *Fli-1* in LPS-induced early inflammatory response in brain pericytes, we knocked down *Fli-1* in cultured mouse brain pericytes using *Fli-1* siRNA. Real-time PCR confirmed *Fli-1* knockdown in cells transfected with *Fli-1* siRNA (Fig. 6A). LPS exposure significantly increased *Fli-1* mRNA levels in brain pericytes at 2 h post-stimulation, but this increase was reversed in cells transfected with *Fli-1* siRNA compared to those with control siRNA (Fig. 6A). Additionally, knockdown of *Fli-1* significantly inhibited the LPS-induced increase in MCP-1 mRNA levels in brain pericytes at 2 h post-LPS stimulation (Fig. 6B). Moreover, IL-6 and MCP-1 protein levels in the supernatants of brain pericytes were markedly elevated 2 h after LPS stimulation, which was suppressed by *Fli-1* knockdown (Fig. 6C-D).

### Inhibition of *Fli-1* persistently suppresses LPS-induced inflammation in cultured brain pericytes

**Fig. 7:**
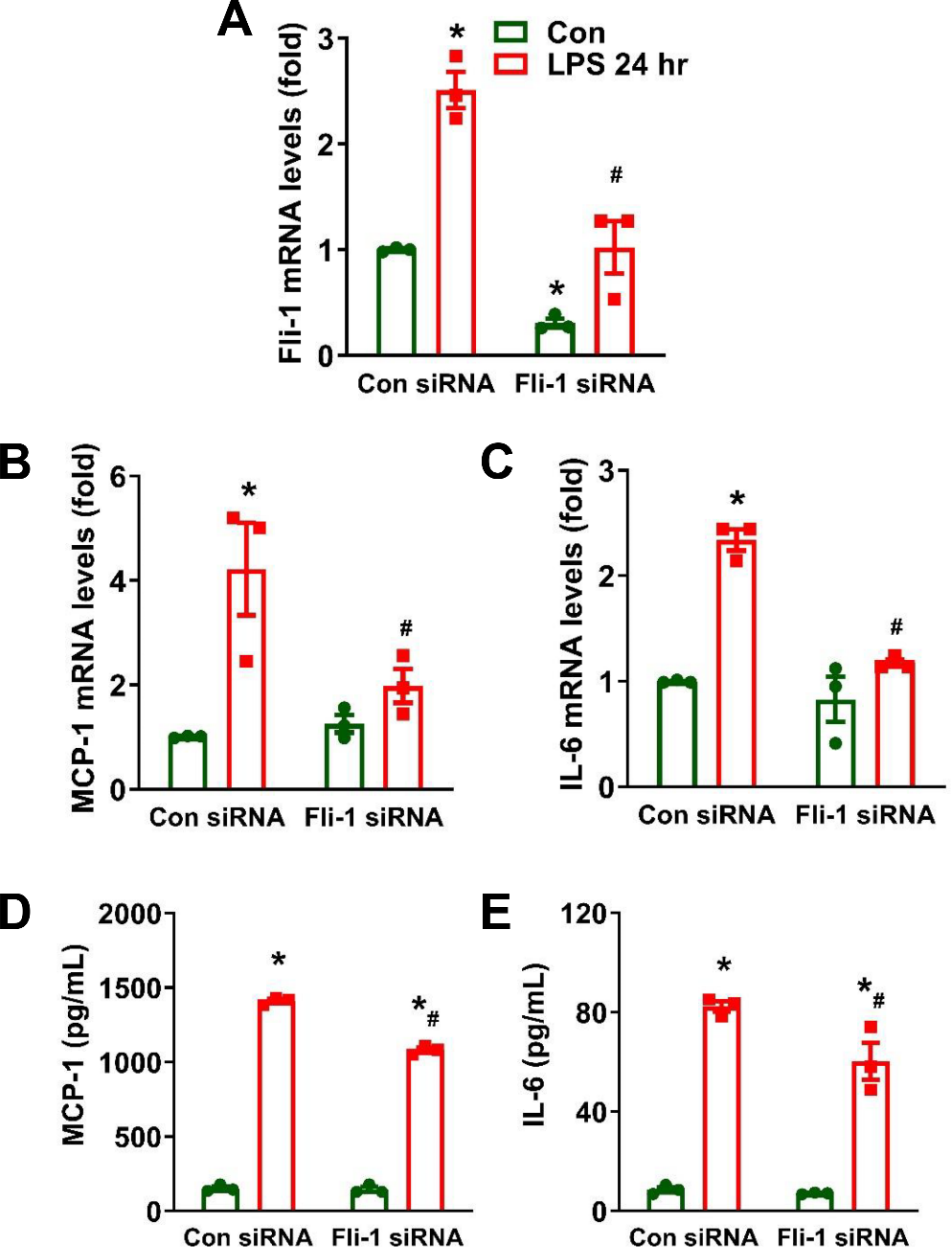
Persistent suppression of LPS-induced inflammation by *Fli-1* knockdown in cultured mouse brain pericytes. Cells were transfected with *Fli-1*-specific small interfering RNA (siRNA) or control siRNA for 24 h and stimulated with LPS (200 ng/mL) for 24 h. mRNA levels of *Fli-1* (**A**), MCP-1 (**B**) and IL-6 (**C**) were measured by Real-time PCR. MCP-1 (**D**) and IL-6 (**E**) levels in the supernatants were quantified by ELISA. *N* = 3 independent experiments. Data are presented as means ± SE. Statistical significance was determined using ANOVA, with *p < 0.05 indicating a significant difference compared to the Control siRNA group and #*p* < 0.05 indicating a significant difference compared to the Control siRNA + LPS group

To further investigate the effect of *Fli-1* knockdown on LPS-induced persistent inflammation in brain pericytes, cells were transfected with either *Fli-1* or control siRNA and then stimulated with LPS for 24 h. As shown in Fig. 7A, *Fli-1* knockdown significantly reduced the LPS-induced increase in *Fli-1* levels. Elevated mRNA levels of MCP-1 and IL-6 at 24 h after LPS stimulation were also decreased in pericytes transfected with *Fli-1* siRNA compared to the control siRNA group (Fig. 7B-C). Interestingly, *Fli-1* inhibition persistently suppressed LPS-induced MCP-1 and IL-6 production in brain pericytes 24 h after stimulation (Fig. 7D-E).

### LPS increases *Fli-1* levels in brain pericytes via TLR4-Myd88 signaling pathway

**Fig. 8:**
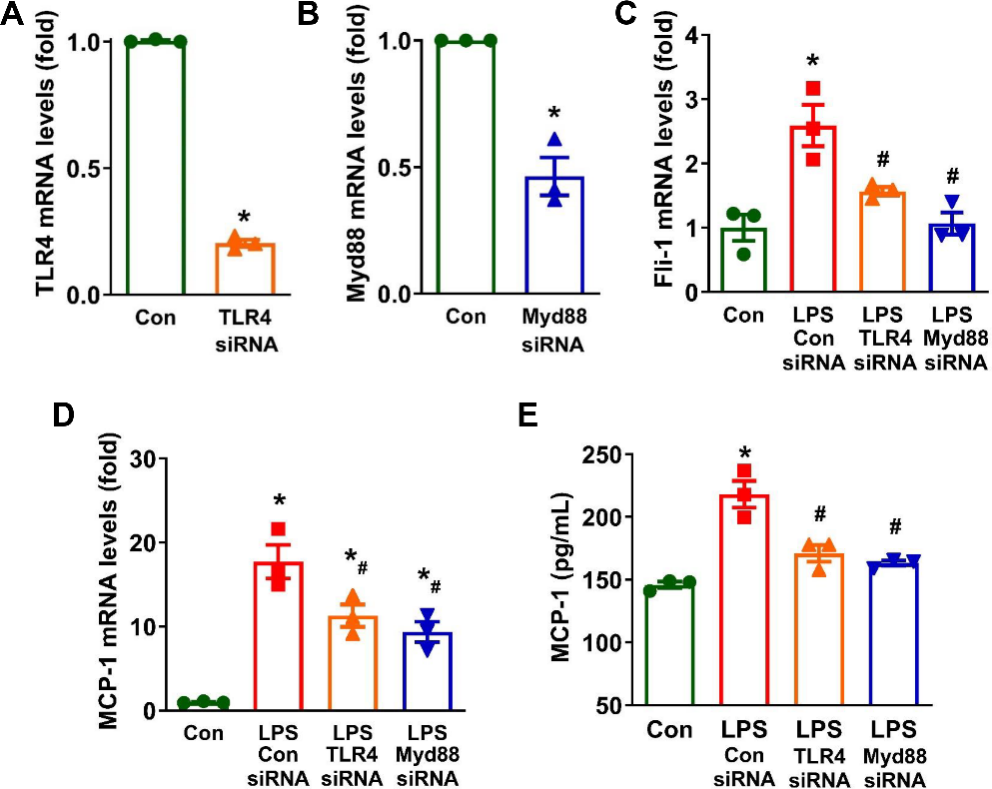
TLR4-Myd88 pathway mediates LPS-induced upregulation of *Fli-1* in mouse brain pericytes. Pericytes were transfected with TLR4 or MyD88 siRNA, or control siRNA, for 24 h followed by LPS stimulation (200 ng/mL) for 2 h. TLR4 (**A**) and MyD88 (**B**) mRNA levels were measured by Real-time PCR. *N* = 3 independent experiments. Data are presented as means ± SE. Statistical significance was assessed using Student’s t-test, with *p < 0.05 indicating a significant difference compared to the Control siRNA group. Additionally, mRNA levels of *Fli-1* (**C**) and MCP-1 (**D**) were determined by Real-time PCR, and MCP-1 protein levels in the supernatants were measured by ELISA (**E**). *N* = 3 independent experiments. Data are presented as means ± SE. Statistical significance was determined using ANOVA, with *p < 0.05 indicating a significant difference compared to the Control group and #*p* < 0.05 indicating a significant difference compared to the Control siRNA + LPS group

The TLR4 signaling pathway has been implicated in the LPS-induced inflammatory response in brain pericytes [25]. To explore the potential link between *Fli-1* and TLR4 signaling, we knocked down TLR4 or Myd88 in cultured mouse brain pericytes using specific siRNAs and then stimulated the cells with LPS for 2 h. The results from Real-time PCR confirmed the successful knockdown of TLR4 or Myd88 (Fig. 8A-B). Inhibition of both TLR4 and Myd88 led to a reduction in the LPS-induced increase in *Fli-1* levels at 2 h post-LPS stimulation (Fig. 8C). Additionally, LPS-induced increases in MCP-1 mRNA levels were suppressed in cells transfected with either TLR4 or Myd88 siRNA (Fig. 8D). Similarly, elevated MCP-1 production in the supernatants of brain pericytes at 2 h after LPS stimulation was also reduced by knockdown of TLR4 or Myd88 (Fig. 8E).

## Discussion

This study reveals several novel and significant findings. First, brain pericytes contribute to the early phase of neuroinflammation during sepsis by producing inflammatory mediators MCP-1. Second, knockout of *Fli-1* in pericytes protects against the early neuroinflammatory response in sepsis. Finally, LPS increases *Fli-1* levels in cultured brain pericytes through the TLR4/MyD88 signaling pathway. Collectively, these results highlight a novel role for brain pericytes in mediating early neuroinflammation during sepsis and underscore the critical role of *Fli-1* in brain pericyte activation. Thus, targeting brain pericyte *Fli-1* may offer a new strategy for addressing initial process of neuroinflammation in sepsis and neuroinflammation-related conditions.

In addition to their known roles in vascular development and homeostasis, pericytes are increasingly recognized for their involvement in the host response to injury, particularly in regulating initial inflammatory process [16]. Hepatic stellate cells (HSCs), which are liver pericytes, play a significant role in LPS-induced liver injury by producing inflammatory mediators such as MCP-1 and IL-6 [26–29]. In the kidney, pericytes contribute to persistent inflammation and development of kidney fibrosis by producing pro-inflammatory mediators after injury, which promotes immune cell infiltration [26]. Similarly, lung pericytes play a crucial role in lung inflammation, exhibiting robust inflammatory responses following lung injury [9, 16]. Recent studies have shown that brain PDGFRβ cells, including pericytes, are activated early during systemic inflammation and act as initial sensors and responders to neuroinflammation by producing MCP-1 [9]. Consistent with these findings, our research further indicates that brain pericytes contribute to the early inflammatory response during sepsis by releasing MCP-1 and IL-6. Given these findings, exploring the role of pericytes in the initial inflammatory process in the brain warrants further investigation.

Our previous research established *Fli-1* as a crucial regulator of the pericyte inflammatory response [9, 14]. Fli-1, a member of the ETS transcription factor family, plays a pivotal role in regulating inflammation by modulating the expression of key cytokines and chemokines, such as MCP-1 and IL-6 [21, 22]. We observed that *Fli-1* knockout in pericytes mitigated sepsis-induced lung injury, and *Fli-1* knockdown in cultured lung pericytes reduced inflammatory responses, indicating that *Fli-1* in lung pericytes contributes to lung inflammation during sepsis [9]. Similarly, we found that *Fli-1* in brain pericyte contributes to the inflammatory response in the brain in Alzheimer’s disease, where inhibition of *Fli-1* reduced inflammatory mediators such as IL-6 in the hippocampus [14]. Consistent with this, we observed that knockout of *Fli-1* in pericytes reduced the early inflammatory response in the brain during sepsis. Additionally, knockdown of *Fli-1* in cultured brain pericytes significantly inhibited LPS-induced production of inflammatory mediators such as MCP-1 and IL-6. MCP-1 plays a critical role in the early stages of inflammation by enhancing the expression of inflammatory factors and recruiting immune cells to sites of inflammation and infection [9, 30, 31]. Previous studies have shown that brain pericytes secrete MCP-1, promoting immune cell infiltration into the brain [32]. Duan et al. reported that brain PDGFRβ cells, including pericytes, are the primary source of MCP-1 during the early phase of neuroinflammation, transmitting signals to the central nervous system [9]. As a transcription factor, Fli-1 activates MCP-1 transcription by binding directly to its promoter [30]. Recent research has shown that *Fli-1* controls MCP-1 expression in liver pericytes [33]. In this study, we further confirmed that elevated levels of *Fli-1* in brain pericytes increase MCP-1 expression, thereby contributing to the early inflammatory response in the brain during sepsis. Therefore, targeting Fli-1 to prevent pericytes activation could potentially reduce initial inflammation in organs affected by inflammatory diseases like sepsis and improve clinical outcomes.

Our previous studies highlighted Fli-1’s direct impact on cytokine and chemokine transcription in pericytes [9, 14]. However, the signaling pathways regulating Fli-1 in pericytes remain largely unexplored. Circulating LPS has been shown to activate the TLR4 signaling pathway, which subsequently increases the levels of inflammatory mediators like MCP-1 in the brain [8, 34]. Additionally, LPS-induced TLR4-Myd88 activation has been linked to inflammatory responses in hepatic stellate cells, the liver pericytes, contributing to liver inflammation and fibrosis [27–29]. LPS also enhances TLR4 expression in rat lung pericytes [35] and promotes inflammatory responses in human brain pericytes through the TLR4 signaling pathway [25]. Based on these findings, we demonstrated for the first time that LPS exposure elevates *Fli-1* levels in mouse brain pericytes via the TLR4/Myd88 signaling pathway. This increase in *Fli-1* further contributes to the early inflammatory response by upregulating inflammatory mediators MCP-1 and IL-6. Thus, activated brain pericytes may play a role in the initial inflammatory response during sepsis through the release of cytokines and chemokines mediated by LPS-induced TLR4/Myd88 signaling, with Fli-1 acting as a crucial regulator in this process.

## Conclusions

In conclusion, our findings underscore the crucial role of brain pericytes in the early inflammatory response during sepsis and reveal a novel function of *Fli-1* in regulating brain pericyte-mediated inflammation. These results highlight that targeting Fli-1 in pericytes could represent a promising therapeutic strategy for sepsis-associated encephalopathy and other neuroinflammatory conditions, warranting further investigation.

## Data Availability

Data is provided within the manuscript or supplementary information files.
